# Supplementary material for: TSA Activates Pluripotency Factors in Porcine Recloned Embryos
Source: Genes (Basel). 2022 Apr 7;13(4):649. doi: 10.3390/genes13040649 (PMC9029504; doi:10.3390/genes13040649)
Supplement: Supplementary file 1 [file genes-13-00649-s001.zip › genes-1644853-supplementary.pdf]

Table S1. Primer sets for the construction of reporter vectors, q-PCR and bisulfite sequencing.

Primer Name	Sequence (5′-3′)	NCBI Reference Sequences
	**For vector constructions**	
pOCT4-F	CTTATTAGACATTGATTATTGACTAGTACAGCAATGCAGGGTGTCTGCAA	CT737281.12
pOCT4-R	CTTGCTCACCATGGTGGCGAATTTGGGGAAGGAAGGCGCCCCAAGC	CT737281.12
pSOX2-F	TAGAACTCGACCACTAGTTAATTAAACACCAACCCCTTGGTTTTTTGTTT	CU914271.8
pSOX2-R	CACCATGGTGGCGAATTCGCTAGCTGCGAGGAAAATCAGACGAAGAATAA	CU914271.8
	**For q-PCR**	
GAPDH-F	GTCGGTTGTGGATCTGACCT	NM_001206359.1
GAPDH-R	GTCCTCAGTGTAGCCCAGGA	NM_001206359.1
OCT4-F	GGTGGAGGAAGCTGACAACA	NM_001113060.1
OCT4-R	TCTCCAGGTTGCCTCTCACT	NM_001113060.1
SOX2-F	GATCAGCATGTACCTCCCCG	NM_001123197.1
SOX2-R	AGGCAGTGTACCGTTGATGG	NM_001123197.1
	**For bisulfite PCR**	
pOUT4-F2 (outer)	TTAGTATAGATATTAGATTTGTGTG	CT737281.12
pOUT4-R2 (outer)	AAACCGAAAACCCAATAAAACCAAA	CT737281.12
pOUT4-F3(inner)	GAAGAGGGGTTTAATATTTGGTTTT	CT737281.12
pOUT4-R3(inner)	CACCCACTAACCTTAACCTCTAAC	CT737281.12
